# Reducing admission time to Broadmoor High Secure Hospital – a case review

**DOI:** 10.1192/bjo.2021.826

**Published:** 2021-06-18

**Authors:** Maria Vittoria Capanna, Saima Ali, Robert Bates

**Affiliations:** 1 West London NHS Trust; 2 Broadmoor Hospital, Crowthorne Berks

## Abstract

**Aims:**

Prolonged waiting times for admission to psychiatric hospital settings are a common and widespread issue. Delayed admissions may result in poorer outcomes due to prolonged mental suffering and delays in initiating treatment. Long waiting times also have a negative impact at a service level, impeding patient flow.

National guidance has been recently updated, recommending that patient transfers to secure services take no longer than 28 days from referral. These transfers are frequently affected by delays in admission, possibly resulting in increased risk to patients, staff and the public.

The aim of this project was to audit all referrals to Broadmoor High Secure Hospital in England within a one year period with special focus taken on calculating the time taken from referral to admission. We aimed to assess if there were any rate limiting steps which could be targeted to reduce time from referral to admission.

**Method:**

We collected data and conducted a retrospective cohort review for all admissions from September 2019-September 2020. Where available, information was obtained for each step of the referrals process. Individual patient records were reviewed where required.

Exclusion criteria: data withdrawn, transfers from other high secure services (HSS), incomplete data, “MOJ instruction” or urgent admission bypassing the process.

**Result:**

18 cases were excluded as per exclusion criteria. 46 patients were included in the study. 16 referrals originated from medium secure psychiatric hospitals, and 30 from prison.

The average time from referral to admission was 44.3 days. Admission of patients from MSUs was quicker, taking an average of 40.3 days when compared to prison referrals, which took 45.9 days.

The breakdown of timings for each step in the referrals process was calculated to determine if a rate limiting step could be identified.On average it took 2.1 working days to allocate a case to a clinician, 7.6 days for an assessment, 9.2 days to complete a report and 3.5 days to submit this to the admissions panel. The mean time from referral to the date of the panel hearing was 22.5 working days, and admission took a further 21.8 days on average.

**Conclusion:**

The current average time to admission exceeds the new 28 day recommendation. This could both be due to the COVID-19 pandemic, and miscommunication about time targets. We will review the process and aim to reduce the time from referral to admission in line with new guidance.

